# Novel evidence for cue-based retrieval of top-down sets in spatial cueing

**DOI:** 10.3389/fcogn.2024.1305382

**Published:** 2024-03-04

**Authors:** Christian Büsel, Christian Valuch, Rebecca Rosa Schmid, Pierre Sachse, Ulrich Ansorge

**Affiliations:** ^1^Department of Psychology, University of Innsbruck, Innsbruck, Austria; ^2^Georg-Elias-Müller-Institut für Psychologie, University of Göttingen, Göttingen, Germany; ^3^Department of Cognition, Emotion, and Methods in Psychology, University of Vienna, Vienna, Austria

**Keywords:** contingent-capture, cueing, memory retrieval, task-sets, visual attention

## Abstract

Task cues that correctly (vs.incorrectly) inform humans about their upcoming tasks, benefit (vs. interfere with) performance because participants can use task cues to retrieve the corresponding task set, so that targets can (vs. cannot) be processed according to the currently applying task set from target onset onwards. Here, we tested if task-associated features of peripheral cues have a similar effect. Typically, peripheral cues with a task-associated, searched-for color (i.e., top-down matching cues) capture attention: Search for targets presented at the cued position (valid condition) is faster than for targets presented away from the cue (invalid condition), even if cues do not predict the likely target location. For example, when searching for red and green targets, a red cue captures attention even if presented prior to a green target, but a blue cue does not. We know that cue-target color congruence—whether the cue has a target-similar color (congruent case) vs. a target-dissimilar color (incongruent case)—additionally expedites vs. delays search times. However, it is unclear if this congruence effect reflects feed-forward color priming of the target only; or if cue-elicited retrieval of color-specific task sets is involved. Crucially, we hypothesized that cue-based task-set retrieval should incur additional costs if the task sets for the two target colors differ more. In contrast, mere feed-forward priming should not be affected by task-set similarity between color-associated task sets. Congruence effects were indeed larger when color-associated task sets were more different. This finding indicates cue-elicited retrieval of color-associated task sets can contribute to effects of cue-target color congruence. Results are discussed in light of recent theories.

## Introduction

Visual input can capture human visuo-spatial attention involuntarily. For example, in peripheral cueing, participants search for a pre-defined target following an uninformative cue at one potential target location. Despite being no help and irrelevant, the cue sometimes captures attention: In valid trials (the cue being presented at the subsequent target position), the cue facilitates target search, compared to invalid trials (the cue being presented away from the subsequent target position). Folk et al. (1992) were the first to demonstrate that the *validity effect*, as evidence of attention capture by the cue, is contingent on top-down task sets participants hold in (visual working) memory (VWM). For example, when participants search for a green target, only task-set matching (i.e., green) cues elicit a validity effect, whereas non-matching (e.g., blue) cues do not. This is the top-down *contingent-capture effect* (for a review, see Büsel et al., 2020).

The contingent-capture effect reflects a dependence of cue-elicited attention capture on memory content. However, does the opposite hold, too? Can peripheral cues elicit retrieval of matching task sets from VWM? Recent theories and findings imply this possibility (Moore and Weissman, 2010, 2011; Frings et al., 2020). However, some results that seemingly support the assumption of cue-elicited task-set retrieval are open to alternative explanations. Take the example of cue-target color congruence effects. When participants search for two colors simultaneously (e.g., red and green), responses are faster when cue and target colors are the same (or congruent) than if not (e.g., Irons et al., 2012; Büsel et al., 2019). In theory, cue-target color-congruence effects could reflect cue-elicited retrieval of color-specific task-sets from VWM (Büsel et al., 2019): the red cue would elicit retrieval of a task set to search for (a) red (target). Hence, participants would have to switch to a different task set to find the target only in cue-target color-incongruent trials (e.g., if a green target follows a red cue) but not in cue-target color-congruent conditions. Such task-set switches are known to create a cost that could account for the color-congruence effect (Monsell, 2003; cf. Kiesel et al., 2010). However, cue-target color congruence effects could likewise reflect feedforward priming of target colors by preceding cue colors (e.g., Irons et al., 2012; Kerzel and Witzel, 2019).

### Major predictions

To test if cue-color congruence effects could reflect cue-elicited task-set retrieval, we compared cue-color congruence effects during two-color (*2C*, with *C* for *Color*) search under two conditions. In one condition, the color-specific task sets associated with the two different searched-for target colors were more similar: here, we instructed participants to respond by an identical “shape”-response mapping to all targets. For instance, participants pressed the right key for a *T* tilted clockwise and the left key for a *T* tilted counter-clockwise, regardless of target color (i.e., green or red). This was the more similar task-set condition (*2CS* condition, with *S* for *Similar*). In an alternative condition, in contrast, response mappings varied between target colors. For example, while green targets required pressing the right key for clockwise and the left key for counter-clockwise rotated *T*s, the response mappings were switched for red targets. These were the more different task-set conditions (*2CD*, with *D* for *Different*). If cue-elicited retrieval of task sets from (VW) memory contributes to the cue-target color-congruence effect, we expected the cue-target color-congruence effect to be larger under 2CD conditions than under 2CS conditions because cue-elicited task-set retrieval from memory would likely cause additional switch costs between different color-specific mappings under cue-target color-incongruent 2CD conditions than under color-incongruent 2CS conditions (cf. Koch and Allport, 2006). In contrast, feedforward cue-target color priming should be equal under 2CS and 2CD conditions.

#### Additional tests

Related to the question if cues elicited task-set retrieval, and to verify if task-set differences in 2CD relative to 2CS task-set conditions increased interference, we also assessed mixing costs (Monsell, 2003; Kiesel et al., 2010). We defined mixing costs as processing delays under two task-set (here: 2C, two-color search) conditions relative to one task-set (here; *1C*, with *C* for *Color*; one target-color search) conditions. Higher mixing costs in 2CD than 2CS conditions should confirm that task-set differences were indeed higher in the former than in the latter condition. Most likely, 2CD conditions should, thus, increase both switching costs or cue-target color-congruence effects *and* mixing costs. However, it is also possible that participants chose a different strategy to handle the costs elicited by different retrieved task sets in the cue-target color-incongruent 2CD conditions. For example, participants might invest more effort in pre-activating two alternative tasks sets prior to the targets and, hence, prior to all cues, too. If this is the case, we might observe mixing costs, but maybe no increased color-congruence effects or switching costs under 2CD compared 2CS conditions. In this sense, investigation of mixing costs is necessary. Mixing costs provide a complementary view on our major research questions and can provide evidence for alternative strategies in case the predicted interactions between cue-target color congruence and task-set similarity are not borne out by the data.

Note that mixing costs needed to be estimated independently from switching costs at the level of the cue-target color relations *and* the target-to-target color relations because switching costs would only occur in two-color (2C) but not one-color (1C) search. The analyses of the mixing costs are, therefore, restricted to those trials of the 2C (two-color search) blocks in which the target color in a current trial *N* was repeated from the preceding trial *N-1*. For the same reason, the analyses of the mixing costs were restricted to only those trials of the 2C (dual-search) blocks in which the color of the top-down matching cue was congruent to that of the target. In other words, in these respects (i.e., the lack of any potential switching costs for relevant colors in the 1C blocks), the analysis of the mixing costs required that corresponding conditions from 1C (one-color) and 2C (two-color) search conditions were compared to one another.

## Experiment

### Methods

#### Participants

Thirty-seven participants participated (21 female; age range: 18–29 years). All were students of the University of Vienna or the University of Innsbruck and participated in exchange for course-credit. Our aim was to collect sufficient data for testing interactions between color-congruence effects and the degree of task-set differences. Based on the size of (within-trial) cue-target color congruence effects in Büsel et al. (2019), 19 participants would be required to find an effect of similar size with a power of 0.90. Due to ongoing hygienic restrictions, not all participants could be tested under laboratory conditions. We decided to roughly double the minimum sample size for detecting cue-target color congruence effects to compensate for different testing environments and test the hypothesized interactions with task-set differences. Overall, 19 participants completed the experiment in the laboratory, and 18 completed the experiment on their personal laptops at home.

#### Apparatus and stimuli

Under laboratory conditions, data collection was conducted in a dimly lit room with the viewing distance fixed at 57 cm using a chin and forehead rest. Participants who completed the experiment at home were sent the experiment file and carefully instructed to only start the experiment when they felt well-rested. In addition, they were asked to dim any light source around them to avoid reflections on the monitor and complete the experiment within 90 min after starting it.

As the precise stimulus colors and -sizes for participants completing the experiment at home are unknown, the color values and -sizes reported here reflect laboratory conditions utilizing the apparatus detailed above. All colors in the laboratory version of this experiment were equiluminant (~15 cd/m^2^). The fixation display consisted of a gray cross at screen center (visual angle: 0.46° × 0.46°). Two circles (outline thickness: 0.11°) with a diameter of 1.38° were presented 1.95° to the left and right of the fixation cross. In the cue and target displays, the outlines of the circles thickened to 0.34°. In the cue display, one of the circles remained gray while the other—the colored cue—could either be blue (*x* = 0.143, *y* = 0.055), green (0.298, 0.581), or red (0.629, 0.330). In the target display, colored and rotated *T*s appeared within gray circles at the same positions as were used in the cue displays. The target, a tilted letter *T*, was either red or green, while the distractor could randomly be cyan (0.233, 0.357) or magenta (0.334, 0.159). The target letter was either tilted clockwise or counter-clockwise, while the distractor letter could be upright, upside down, tilted clockwise, or tilted counter-clockwise. The distractor letter had never the same orientation as the target letter.

#### Procedure

**Figure 1:**
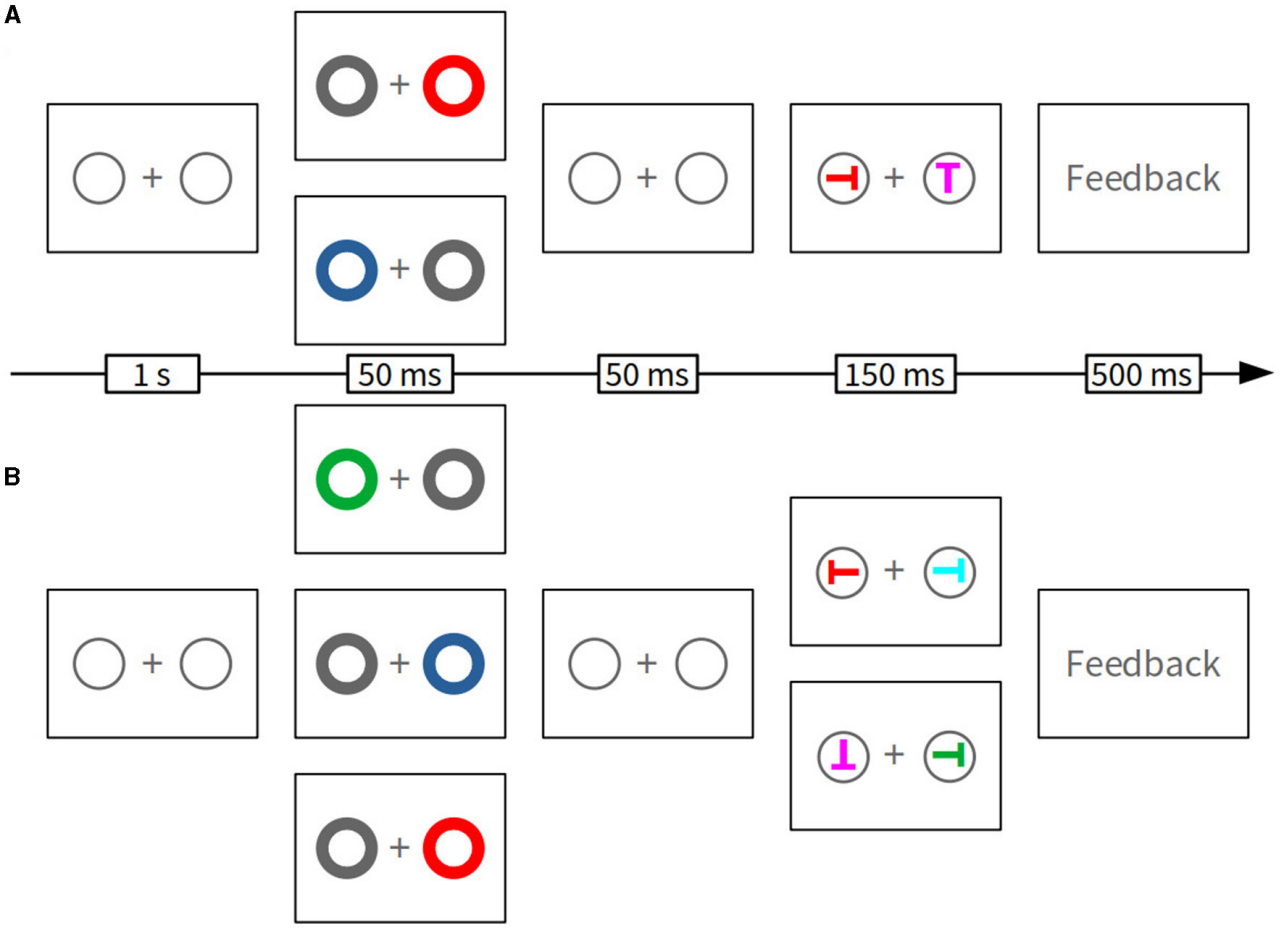
Illustration of the trial sequence in one-color search **(A)** and two-color search conditions **(B)**. In **(A, B)**, boxes depicted second from left illustrate examples of alternative cue displays. Only in **(B)**, boxes second from right depict examples of alternative target displays with alternative target colors (red vs. green). Note that the stimuli are not drawn to scale.

Exemplary trials are illustrated in Figure 1. At the beginning of each trial, the fixation display was presented for 1 s, followed by the cue display for 50 ms. The cue did not predict the correct target identity or position because it was presented at the correct target location on only 50% of trials. Red and green cues served as top-down matching cues and were used with equal frequency, each in a quarter of all trials, whereas blue cues served as top-down non-matching cues and were used in the other half of the trials. Cue and target colors were uncorrelated across trials. We informed participants about all of this and instructed them to ignore the cue. Another fixation display followed for 50 ms, and then the target display was presented for 150 ms. Optional feedback was presented for 500 ms if participants committed an error (“Wrong;” German: “Falsch”), if reaction time (RT) exceeded 1,200 ms (“Respond faster!” German: “Schneller antworten!”), or if participants did not respond within 2 s (“Too slow;” German: “Zu langsam”).

Participants started the experiment with the *two-color search/similar task-set* (2CS) block. We used this fixed order of two-color search blocks before *one-color search blocks* (see below) to prevent known transfer effects: biases in the two-color blocks to attend to colors previously used in a one-color search block (cf. Ansorge et al., 2021). Participants searched for the target, which was equally likely red or green. Participants responded to the orientation of the *T* inside of the target. If the *T* was tilted counter-clockwise, participants pressed the *y*-key, and if the target was tilted clockwise, participants pressed the *m*-key.

After the first block, participants completed the *two-color search/different task-sets* (2CD) block. Displays and conditions were identical to the 2CS block, with one exception: whereas the response mapping for one target color remained the same as in the 2CS block, the response mapping of the other target color reversed (*y-*key for *T*s tilted clockwise and *m*-key for *T*s tilted counter-clockwise). The sequence of 2CS before 2CD block was also kept constant so as to allow general learning effects to transfer from the first to the second block and, thus, if anything, to counteract the predicted slowing under 2CD relative to 2CS conditions. Across participants, we counterbalanced which color target's response mapping changed.

At the end of the experiment, participants completed two *one-color search* (henceforth: 1C) blocks, in which the target color (i.e., red or green) was fixed throughout the block. The order of target colors in the last two blocks was counterbalanced across participants. Target-response mapping in these two blocks was identical to the target-response mapping in the 2CS block.

Forty practice trials preceded each of the *two-color search* blocks, while 10 practice trials preceded each of the last two blocks (*one-color search* blocks). Self-paced breaks were possible following every 100 trials.

#### Design

Different cue and target positions, target colors (where it applied, i.e., in the *two-color search* blocks), and target orientations were equally frequent and varied orthogonally to one another. Within each block, the resulting trials were presented in a pseudo-randomized sequence. To test for contingent-capture effects, we analyzed performance in the two *two-color search* blocks, with the within-participant variables *validity* (valid; invalid) and *cue type* (top-down matching; non-matching). Note that there were no task-set associations for the non-matching cues. In addition, the non-matching cues were never color-congruent to the targets. For the latter reason, they could not be used in a fully factorial design of the conditions with top-down matching cues that we, therefore, tested in a separate analysis presented next.

To investigate cue-elicited memory retrieval, we analyzed only the top-down-matching conditions of the 2C blocks, with the within-participant variables *validity* (valid; invalid), *cue-target color congruence* (henceforth shortened to *congruence*; congruent: cue color = target color; incongruent: cue color ≠ target color), *trial-by-trial target-color repetition* (henceforth shortened to *repetition*; repetition: *N-1* target color = *N* target color; switch: *N-1* target color ≠ *N* target color), and *task-set similarity* (2CS; 2CD). Note that it was not possible to include one-color (1C) search blocks in this analysis because there were no top-down matching/color-incongruent conditions in the one-color search blocks and because there were no trial-by-trial target color switch conditions in the one-color search blocks. Note also that there were no cue-target color congruent conditions with the non-matching cues. For these reasons, the analysis aiming to test the interaction between validity and task-set similarity of the task-associated (top-down matching) cues was conducted without one-color search block performance and without non-matching cueing conditions. An additional analysis concerned mixing costs in the two-color search relative to one-color search conditions of only the *trial-by-trial target-color repetition* trials, with the single variable *search type* [one-color search (1CS); two-color search/similar task-sets (2CS); two-color search/dissimilar task-sets (2CD)]. This analysis was conducted with only trial-by-trial target color repetitions from the two-color search blocks because we wanted to measure the mixing cost independently of the switch cost (cf. Kiesel et al., 2010). In addition, the one-color search blocks do not have trial-by-trial target color switches at all. Because the same is true of the cue-target color incongruent conditions (i.e., they were only realized in two-color search but not one-color search conditions), the analysis used only the cue-target color-congruent conditions of the two-color search blocks.

Aiming for at least 25 measurements per combination of the mentioned within-participant variables, each of the two *two-color search* blocks consisted of 400 trials. The two *one-color search* blocks (that were added to allow for the calculation of mixing costs) followed a 2 × 2 design, with the variables *validity* (valid; invalid) and *cue match* (matching; non-matching), consisting of 100 trials each. In total, the experiment consisted of 1,000 trials (excluding practice trials), and it took the participants about 1 h to complete.

## Results

Data from two participants were excluded because their error rates deviated more than 2.5 *SD*s from the mean error rates. Furthermore, response times below 200 ms or above 1,200 ms were excluded (3.33%). Only correct trials were used in response time analyses.

### Contingent-capture effects

First, before investigating peripheral cue-based task-set retrieval with the help of different vs. similar response mappings, it is paramount to ensure that our response mapping manipulation does not alter the known pattern of results in the contingent-capture protocol (i.e., contingent-capture effects). Hence, to check whether top-down guided search performances (i.e., contingent-capture effects) are comparable under 1C, 2CS, and 2CD conditions, we first ran a mixed analysis of variance (ANOVA), with the within-participant variables *validity* (valid; invalid), *cue match* (matching; non-matching), and *condition* (1C; 2CS; 2CD). To note, this analysis collapsed across different levels of the independent variables *trial-by-trial target color repetition* and of *cue-target color congruence* that were later used in complementary analyses because these two independent variables were only realized in 2C conditions.

To control for additional variance in our data, we also included the between-participants variable of *test environment* (laboratory; home). We mainly focused on the two-way interaction between validity and cue type and tested whether this interaction was further modulated by task-set similarity.

#### Response times

**Figure 2:**
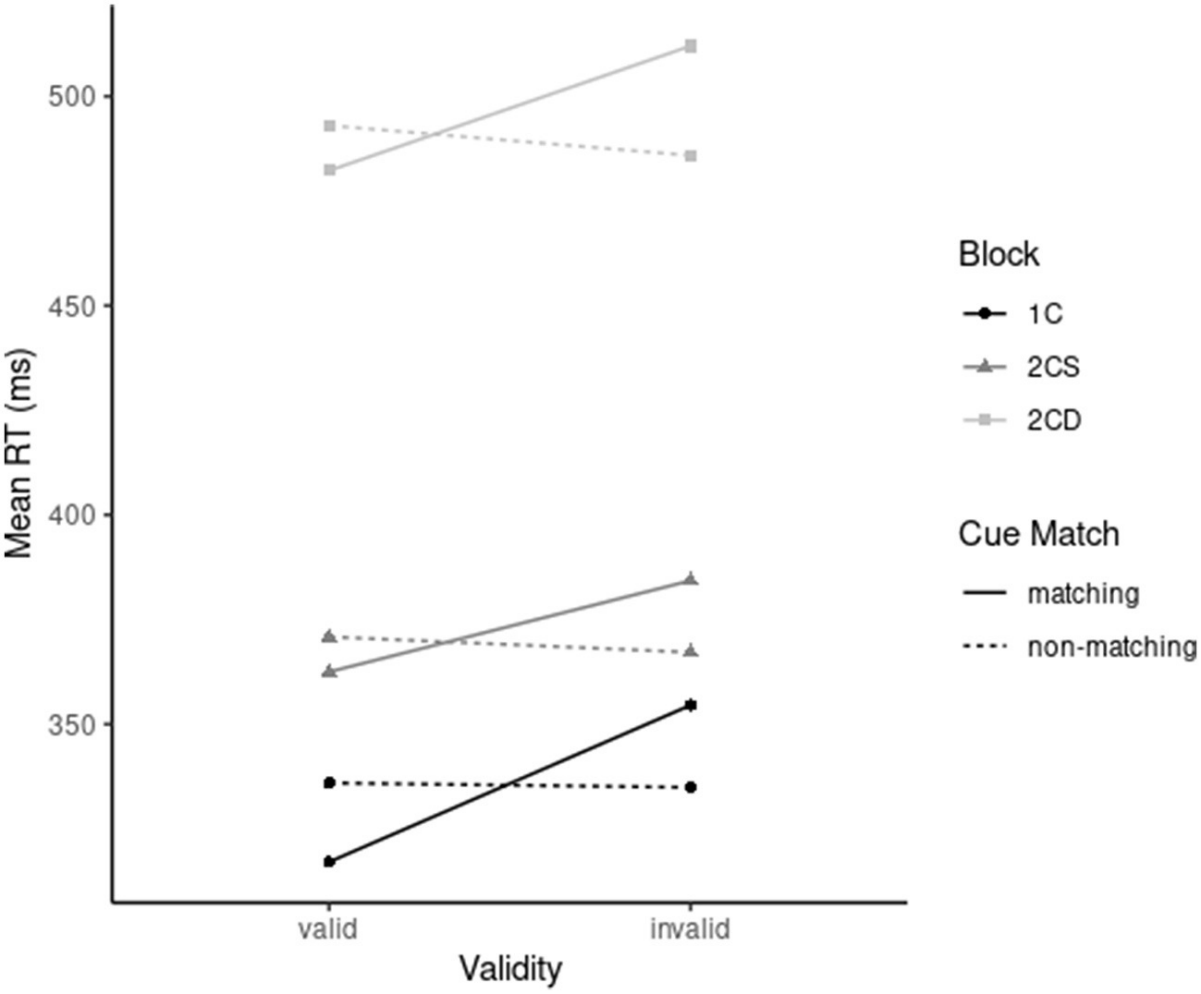
Mean response time (RT, on the *y*-axis) as a function of validity (*x*-axis), cue match [solid lines: matching (i.e., red or green cue); dashed lines: non-matching (i.e., blue cue)], and condition [light gray: two-color search/different task-sets (2CD); darker gray: two-color search/similar task-sets (2CS); black: one-color search (1C)]. The analysis revealed additive effects of contingent-capture and condition, represented by an absence of a significant interaction between these three variables (*p* = 0.113).

The main effects of validity, cue match, and condition were significant, with *F*_(1, 33)_ = 54.66, *p* < 0.001, $\eta_{\text{p}}^{2}$ = 0.62, *F*_(1, 33)_ = 7.30, *p* = 0.011, $\eta_{\text{p}}^{2}$ = 0.18, and *F*_(2, 33)_ = 465.77, *p* < 0.001, $\eta_{\text{p}}^{2}$ = 0.93, respectively. Overall, a validity effect of 12 ms was found and non-matching cues led to 5 ms faster responses than matching cues. Regarding the main effect of condition, participants mean RT was 334 ms in 1C, 370 ms in 2CS, and 495 ms in 2CD conditions (all differences *p* < 0.001). The two-way interaction between validity and cue match was significant, with *F*_(1, 33)_ = 121.08, *p* < 0.001, $\eta_{\text{p}}^{2}$ = 0.79. This interaction was due to a significant validity effect for matching cues, 29 ms, *t*_(34)_ = 11.03, *p* < 0.001, *Cohen's d*_*z*_ = 0.63, and a numerically small but significant inverted validity effect for non-matching cues, −4 ms, *t*_(34)_ = −2.07, *p* = 0.046, *d*_*z*_ = −0.1. Critically, the three-way interaction between validity, cue match, and condition was not significant (*p* = 0.113; see Figure 2).

#### Error rates

We repeated the same analysis on arcsine transformed error rates. Again, the interaction between validity and cue match was significant, with *F*_(1, 33)_ = 23.15, *p* < 0.001, $\eta_{\text{p}}^{2}$ = 0.41. This time, however, the three-way interaction between all variables was significant as well, with *F*_(2, 66)_ = 3.87, *p* = 0.026, $\eta_{\text{p}}^{2}$ = 0.11. *Post-hoc* paired *t*-tests were performed to examine this latter interaction. Under 1C conditions, significant validity effects were found under matching cue conditions, valid: 2.5% vs. invalid: 6.1%, *t*_(34)_ = 5.36, *p* < 0.001, *d*_*z*_ = 1.11. No validity effects were found for non-matching cues (*p* = 0.51). Under 2CS conditions, significant validity effects were found under matching cue conditions, valid: 4.2% vs. invalid: 7.4%, *t*_(34)_ = 3.84, *p* < 0.001, *d*_*z*_ = 0.79. The reverse pattern was found for non-matching cues, valid: 7.2% vs. invalid: 6.1%, *t*_(34)_ = −2.15, *p* = 0.039, *d*_*z*_ = −0.24. Finally, under 2CD conditions, no such validity effects were detected (both *p*s > 0.6).

In conclusion, the three-way interaction indicated that behavior across search conditions was relatively similar under matching conditions, but, here, the validity effect was weaker (i.e., not significant) under 2CD conditions. Slightly other differences in validity effects were found under non-matching conditions. Here, only in the 2CS conditions, an inverse validity effect was observed, possibly reflecting more object-updating costs or active suppression of the non-matching cues in this search condition (cf. Carmel and Lamy, 2014; Schoeberl et al., 2018). We do not have an explanation for these performance differences, but want to emphasize that these differences are not too worrying for our major research question that concerned the top-down matching cueing conditions.

### Congruence and repetition effects

We ran mixed ANOVAs, with the within-participant variables *validity* (valid; invalid; cue-target color) *congruence* (congruent; incongruent; trial-by-trial target color) *repetition* (repetition; switch), and *task-set similarity* (2CS; 2CD). This analysis was restricted to the data of the two-color search blocks because the different levels of the independent variables of cue-target congruence and trial-by-trial target color repetition were not all realized in one-color search blocks. This analysis also used only top-down matching cues because there were no congruent conditions under non-matching conditions. Like in our analysis of contingent-capture effects above, we also included the between-participants variable of *test environment* (laboratory; home). To keep results concise, we report effects and interactions of test environment in the Appendix A1 because these did not alter the conclusions regarding our hypotheses. Identical analyses were calculated with arcsine-transformed error rates.

#### Response times

All condition means are listed in Table 1. Significant main effects were found for validity, *F*_(1, 33)_ = 81.43, *p* < 0.001, $\eta_{\text{p}}^{2}$ = 0.71, congruence, *F*_(1, 33)_ = 157.22, *p* < 0.001, $\eta_{\text{p}}^{2}$ = 0.83, repetition, *F*_(1, 33)_ = 98.13, *p* < 0.001, $\eta_{\text{p}}^{2}$ = 0.75, and task-set similarity, *F*_(1, 33)_ = 486.31, *p* < 0.001, $\eta_{\text{p}}^{2}$ = 0.94. Participants were 26 ms faster in valid compared to invalid trials. Congruent cues sped up responses by 41 ms compared to incongruent cues. Trial-by-trial target-color repetition sped up responses by 33 ms compared to switches (i.e., target-to-target switch costs). Finally, participants were 126 ms faster in the 2CS condition than the 2CD condition. Note that, since we used a fixed block order, this difference may be even larger than reported here because participants were more familiar with the experimental procedure in 2CD blocks.

**Table 1:** Mean response times in each condition in milliseconds.

**Target-color repetition**	**Validity**	**Cue-target congruence**		
		**Congruent**	**Incongruent**	Δ
**2CS**				
Repetition	Valid	345	371	26
	Invalid	374	390	16
Switch	Valid	358	376	18
	Invalid	377	396	19
**2CD**				
Repetition	Valid	423	486	63
	Invalid	458	505	47
Switch	Valid	475	550	75
	Invalid	507	580	73

T-tests were not calculated since the four variables did not significantly interact in the analysis of variance. For better readability, the between-participants variable of test environment was dropped in this table.

Significant two-way interactions were found between repetition and task-set similarity, *F*_(1, 33)_ = 82.95, *p* < 0.001, $\eta_{\text{p}}^{2}$ = 0.72, and between congruence and task-set similarity, *F*_(1, 33)_ = 45.34, *p* < 0.001, $\eta_{\text{p}}^{2}$ = 0.58. However, all three variables included in these interactions also interacted with each other in a three-way interaction, with *F*_(1, 33)_ = 4.84, *p* = 0.035, $\eta_{\text{p}}^{2}$ = 0.13. Hence, we only report *post-hoc* tests for this higher order interaction.

**Figure 3:**
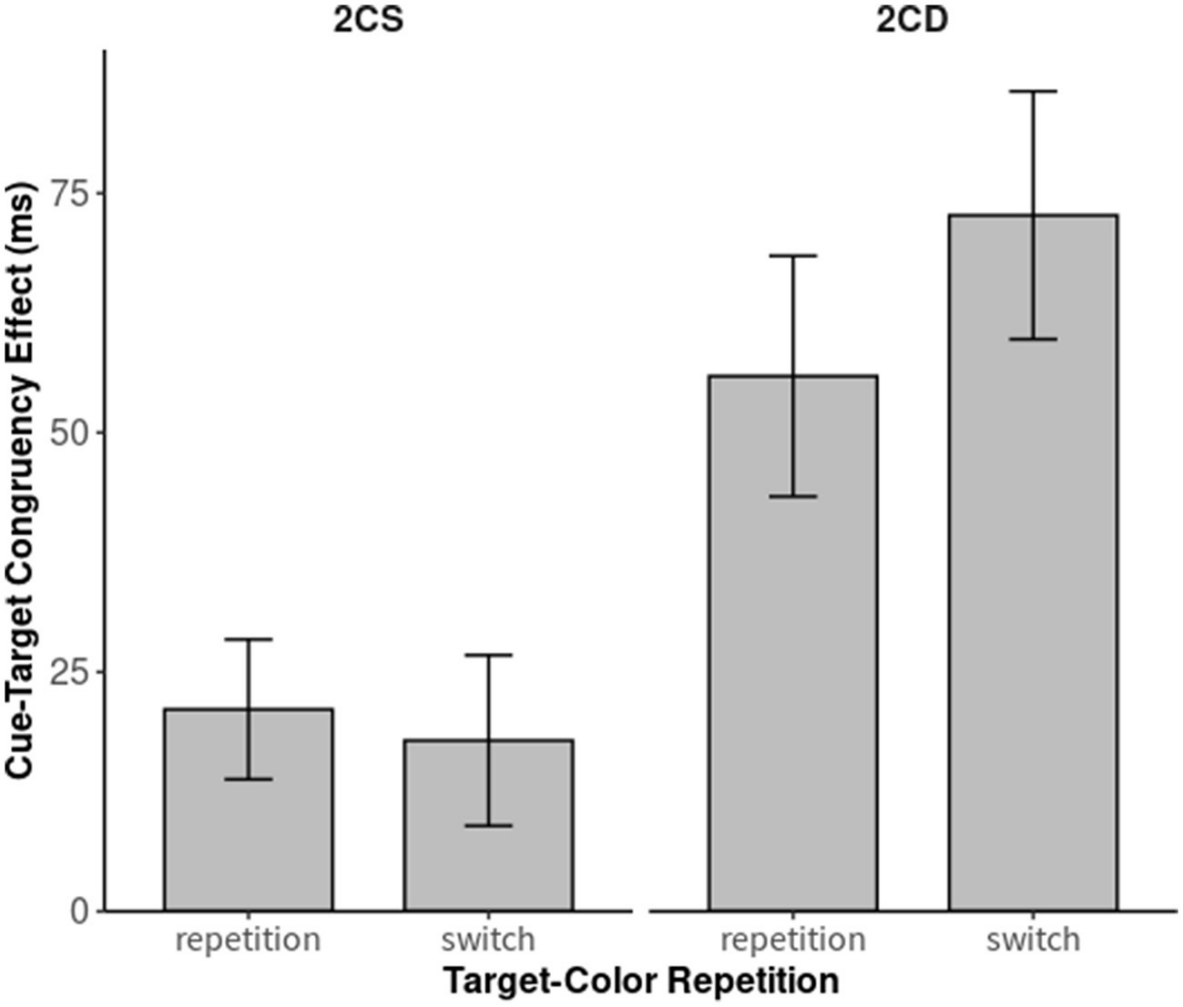
Cue-target congruence effects (i.e., response times, RTs: *RTs*_*incongruent*_ –* RTs*_*congruent*_) as a function of target-color repetition in both the two-color search/similar task-sets [2CS; **(left)**] and two-color search/different task-sets (2CD) conditions **(right)**. We defined cue-target color congruence by the identity vs. non-identity between top-down matching cue and target color within one trial. Error bars indicate the 95% *CI*.

For the *post-hoc* analyses, we split the data according to task-set similarity and ran two additional mixed ANOVAs, including the two within-participant variables (cue-target color) *congruence* (congruent; incongruent) and (trial-by-trial target color) *repetition* (repetition; switch). Again, we also included the between-participants variable of *test environment* (laboratory; home). Concerning the more similar task-set conditions, the 2CS blocks, the two-way interaction of target-color repetition and (within-trial) cue-target color congruence failed to reach significance (*p* = 0.65). Cue-target color congruence effects were significant and of approximately similar size in both target-color repetition trials, 21 ms, *t*_(34)_ = 5.62, *p* < 0.001, *d* = 0.44, as well as target-color switch trials, 18 ms, *t*_(34)_ = 3.98, *p* < 0.001, *d* = 0.38. The same ANOVA on RTs in 2CD conditions, however, revealed a significant interaction between target-color repetition and (within-trial) cue-target color congruence, *F*_(1, 33)_ = 6.76, *p* = 0.014, $\eta_{\text{p}}^{2}$ = 0.17: the congruence effect in target-color repetition trials was much weaker, 56 ms, *t*_(34)_ = 8.84, *p* < 0.001, *d* = 1.02, than in target-color switch trials, 72 ms, *t*_(34)_ = 10.79, *p* < 0.001, *d* = 0.95 (*s*ee also Figure 3). Most critically for our hypotheses, both of the cue-target color congruence effects under 2CD conditions were significantly larger that the cue-target color congruence effects under 2CS conditions. As shown by paired *t*-tests cue-target color congruence effects in trial-by-trial target-color repetition trials were 36 ms larger under 2CD conditions than under 2CS conditions, *t*_(34)_ = 5.21, *p* < 0.001, *d* = 1.2. This difference was even more pronounced in trial-by-trial target-color switch trials, where cue-target color congruence effects were 54 ms larger in 2CD trials than in 2CS trials, *t*_(34)_ = 6.44, *p* < 0.001, *d* = 1.62.

Turning now to trial-by-trial target color repetition effects (or target-to-target switch costs), *post-hoc* paired *t*-tests revealed significant repetition effects that also varied considerably as a function of congruence and task-set similarity. In 2CS conditions, switch costs were found with congruent cues, 10 ms, *t*_(34)_ = 2.29, *p* = 0.028, *d* = 0.21, but not with incongruent cues (6 ms; *p* = 0.24). In 2CD conditions, switch costs were found with congruent cues, 55 ms, *t*_(34)_ = 8.53, *p* < 0.001, *d* = 0.81. However, switch costs with incongruent cues were even larger, 71 ms, *t*_(34)_ = 11.55, *p* < 0.001, *d* = 1.14.

To account for the huge reaction-time differences between 2CS and 2CD conditions, we also calculated relative effects (e.g., Conci et al., 2019). To this end, we calculated individuals' respective congruence effects under all task-set similarity and trial-by-trial target-color repetition conditions and divided them by the subjects mean reaction time from these conditions. Individuals' relative effects were fed into a repeated-measurements ANOVA, with the independent variables *task-set similarity* (2CS; 2CD) and *trial-by-trial target-color repetition* (repetition; switch). The most important finding was replicated: A main effect was found for task-set similarity, with *F*_(1, 34)_ = 30.98, *p* < 0.001, $\eta_{\text{p}}^{2}$ = 0.48. In 2CS blocks, the relative congruence effect was 5.2%, while it was considerably larger in 2CD blocks, with 13.1%. Here, trial-by-trial target color repetition or the interaction between the two variables did not result in a significant effect (*p*s > 0.18).

#### Error rates

We found significant main effects of validity, *F*_(1, 33)_ = 10.88, *p* = 0.002, $\eta_{\text{p}}^{2}$ = 0.25 (cue-target color) congruence, *F*_(1, 33)_ = 5.10, *p* = 0.031, $\eta_{\text{p}}^{2}$ = 0.13 (trial-by-trial target-color) repetition, *F*_(1, 33)_ = 17.98, *p* < 0.001, $\eta_{\text{p}}^{2}$ = 0.35, and task-set similarity, *F*_(1, 33)_ = 41.37, *p* < 0.001, $\eta_{\text{p}}^{2}$ = 0.56. Furthermore, significant two-way interactions between congruence and repetition, *F*_(1, 33)_ = 7.44, *p* = 0.01, $\eta_{\text{p}}^{2}$ = 0.18, and repetition and task-set similarity, *F*_(1, 33)_ = 16.74, *p* < 0.001, $\eta_{\text{p}}^{2}$ = 0.34, emerged. However, all within-participant variables entered a four-way interaction between all within-participant variables, with *F*_(1, 33)_ = 10.92, *p* = 0.002, $\eta_{\text{p}}^{2}$ = 0.25. Hence, we examined cue-target color congruence effects in error rates as a function of task-set similarity, trial-by-trial target-color repetition, and validity. Under 2CS conditions, we only found benefits of cue-target congruence in valid target-color repetition trials (congruent: 3.2% vs. incongruent: 5.2%), *t*_(34)_ = 2.21, *p* = 0.034, *d* = 0.52. In 2CD trials, congruence effects were present in invalid target-color repetition trials (congruent: 6.5% vs. incongruent: 10.4%), *t*_(34)_ = 3.85, *p* < 0.001, *d* = 0.72 (all other *p*s < 0.1; see Table 2).

**Table 2:** Mean cell accuracies.

**Target-color repetition**	**Validity**	**Cue-target congruence**		***t*_(34)_**	***p***	***d***
		**Congruent**	**Incongruent**			
**2CS**						
Repetition	Valid	3.2	5.2	2.21	0.024	0.52
	Invalid	8.7	6.6	−2.21	0.034	−0.39
Switch	Valid	5.3	4	−1.28	0.208	−0.24
	Invalid	6	7.5	0.04	0.966	>0.01
**2CD**						
Repetition	Valid	6.4	7.3	1.9	0.065	0.45
	Invalid	6.5	10.4	3.85	< 0.001	0.73
Switch	Valid	10.5	16.7	1.28	0.147	0.32
	Invalid	13.9	12.8	−1.46	0.154	−0.25

Since all four variables interacted, the test statistics are reported as well.

### Mixing costs (two-color search vs. one-color search conditions)

Analyses were restricted to top-down matching conditions because these were the conditions of interest to see if participants might have at least needed more time to process the task-relevant stimuli under two-color search than one-color search conditions, and under 2CD than under 2CS conditions. This analysis was not strictly necessary after the analyses above provided evidence for congruence effects and congruence effect differences between 2CS and 2CD conditions. However, we had planned the analysis of mixing costs from the start, and we also wanted to ensure that mixing costs supported our interpretations and did not now show less evidence of higher demands in two-color than one-color search or under 2CD than under 2CS conditions. To equate compared conditions across one-color search and two-color search blocks, only trial-by-trial target-color repetition and cue-target color congruent trials of the two two-color search (2CS, 2CD) blocks were included in this analysis.

#### Response times

We found significant mixing costs in the 2CS condition (relative to the one-color search, 1C, conditions), 24 ms, *t*_(34)_ = 4.74, *p* < 0.001, *d* = 0.6, and even higher mixing costs in the 2CD condition, 106 ms, *t*_(34)_ = 16.75, *p* < 0.001, *d* = 2.28. These mixing costs differed significantly, *t*_(34)_ = 14.14, *p* < 0.001, *d* = 2.44.

#### Error rates

Mixing costs were found neither in the 2CS condition, nor in the 2CD condition (*p* > 0.14).

### Supplementary analysis: cue-target color congruency and non-matching cues

Effects of cue-target color congruence based on mere color priming and of cue-target color congruence based on task-set retrieval can also be discriminated from one another by comparing if (1) incongruent top-down matching cues lead to stronger interference than incongruent but non-matching cues because only the former but not the latter are associated with alternative task sets than the target (i.e., non-matching cues are not associated with any task set). It can also be tested if (2) congruent top-down matching cues facilitate processing relative to incongruent non-matching cues. Again, this is possible because only the top-down matching congruent cues could expedite search times beyond color priming by additionally allowing to retrieve the correct target-response mapping even prior to the target.

To this end, we replaced the independent variable (within-trial) *cue-target congruence* of the two-color search block with the three-step variable *cue type* (matching-congruent, matching-incongruent, non-matching). Matching-congruent cues were trials in which cue and target had the same color. In contrast, matching-incongruent cues were trials in which the cue was top-down matching but had a different color than the target. Non-matching cues were blue, meaning that they did not share any features with the searched-for target.

In addition to the independent variable cue type, we fed the factors *validity* (valid, invalid; trial-by-trial target-color) *repetition* (switch, repetition), and *task-set similarity* (2CS, 2CD) into the repeated measurements ANOVAs, both for reaction times and error rates. For the purpose of briefness, we only discuss the relevant main effects of cue type and its interactions here. The complete results can be found in the Appendix A2.

#### Response times

The main effect of cue type was highly significant, with *F*_(2, 68)_ = 116.79, *p* < 0.001, $\eta_{\text{p}}^{2}$ = 0.77. The mean reaction time for trials with non-matching cues was 429 ms, for trials with matching-congruent cues, it was 415 ms, and, for trials with matching-incongruent cues, it was 455 ms (all differences *p* < 0.001). This finding highlights that the shift between task sets in incongruent trials with matching cues impaired performance in comparison with non-matching cues. Furthermore, replicating results found in the analyses above, cue type interacted with validity, *F*_(2, 68)_ = 32.63, *p* < 0.001, $\eta_{\text{p}}^{2}$ = 0.49, repetition, *F*_(2, 68)_ = 32.63, *p* < 0.001, $\eta_{\text{p}}^{2}$ = 0.49, and task-set similarity, *F*_(2, 68)_ = 32.63, *p* < 0.001, $\eta_{\text{p}}^{2}$ = 0.49.

First, examining the interaction between cue type and validity, we found significant validity effects both for matching-congruent cues, 29 ms, *t*_(34)_ = 8.21, *p* < 0.001, *d* = 0.55, and matching-incongruent cues, 23 ms, *t*_(34)_ = 6.33, *p* < 0.001, *d* = 0.47. In contrast, we found an inverted but weak validity effect for non-matching cues, −5 ms, *t*_(34)_ = −2.17, *p* = 0.037, *d* = −0.10. The interaction between cue type and repetition was due to significant but differently pronounced target-to-target switch costs in all cue conditions: 30 ms in matching-congruent cue trials, *t*_(34)_ = 7.53, *p* < 0.001, *d* = 0.58, 37 ms in matching-incongruent cue trials, *t*_(34)_ = 8.47, *p* < 0.001, *d* = 0.73, and 41 ms in non-matching cue trials, *t*_(34)_ = 10.73, *p* < 0.001, *d* = 0.76. This finding underlines that, if anything, matching cue-target congruent trials counteracted target-color switch effects between trials the most, an effect that must be due to the matching cue colors' task associations that were not at work with non-matching cues and that worked in favor of the incorrect task set in the case of a matching-incongruent cue. Finally, concerning the interaction between cue type and task-set similarity, we first looked at trials from the 2CS condition. Here, matching-congruent cues sped up responses by 20 ms compared to matching-incongruent cues, *t*_(34)_ = 6.38, *p* < 0.001, *d* = 0.44. Matching-incongruent cue trials also delayed responding relative to non-matching cue trials by 15 ms, *t*_(34)_ = 5.31, *p* < 0.001, *d* = 0.32. Clearly, this delay must have reflected task-set retrieval by the matching-incongruent cues that was missing for non-matching cues. The reaction time difference between non-matching and matching-congruent cue trials was not significant (*p* = 0.067). Under 2CD conditions, all differences were significant: matching-congruent cues sped up responses compared to matching-incongruent cues [65 ms; *t*_(34)_ = 11.42, *p* < 0.001, *d* = 1.04] and non-matching cues [25 ms; *t*_(34)_ = 7.15, *p* < 0.001, *d* = 0.38]. Again, matching-incongruent cues delayed responding relative to non-matching cues, this time by 40 ms, *t*_(34)_ = 9.2, *p* < 0.001, *d* = 0.63.

#### Error rates

As in reaction times, the main effect of cue type was significant, with *F*_(2, 68)_ = 35.93, *p* < 0.001, $\eta_{\text{p}}^{2}$ = 0.51. Participants committed 8.3, 9.5, and 7.3% errors in trials with non-matching, matching-incongruent, and matching-congruent cues, respectively (all differences: *p* < 0.01). Cue type entered two-way interactions with validity, *F*_(2, 68)_ = 6.35, *p* = 0.003, $\eta_{\text{p}}^{2}$ = 0.16, and target-color repetition, *F*_(2, 68)_ = 5.62, *p* = 0.006, $\eta_{\text{p}}^{2}$ = 0.14. However, all variables included in this analysis entered a four-way interaction, *F*_(2, 68)_ = 6.27, *p* = 0.003, $\eta_{\text{p}}^{2}$ = 0.16, which is why we focus on this latter interaction here and evaluated cue-target congruence effects under different task-set similarity, validity, and target-to-target switch or repetition conditions. In 2CS blocks, under valid trial-by-trial target-color repetition conditions, all cue types led to the following ERs: 7.9% for non-matching cues, 5.2% for matching-incongruent cues, and 3.2% for matching-congruent cues (all differences *p* < 0.05). In valid trial-by-trial target-color switch trials, matching-congruent cues (5.3%) and matching-incongruent cues (4%) led to smaller ERs than non-matching cues (6.4%; both *p*s < 0.001). However, the difference between matching-congruent cues and matching-incongruent cues was not significant (*p* = 0.2). In the remaining conditions of 2CS blocks, all cue types led to similar ERs (*p*s > 0.05). Next, looking at 2CD blocks with valid cues in trial-by-trial target-color repetition trials, matching-congruent cues (6.5%) and matching-incongruent cues (7.28%) differed significantly from non-matching cues (7.32%; both *p*s < 0.03), although this latter difference likely stems from unequal variances. In switch trials with valid cues, only the difference between matching-congruent cues (10.4%) and non-matching cues (12.1%) reached significance (*p* < 0.01). In invalid trial-by-trial target-color repetition trials, the differences between matching-congruent (6.5%) and matching-incongruent cues (10.2%), and between matching-congruent and non-matching cues (6.8%) was significant (both *p*s < 0.01). Finally, in 2CD trial-by-trial target-color switch trials with invalid cues, the difference between matching-incongruent cues (12.8%) and non-matching cues (13.6%) was significant (*p* = 0.01). These interactions are difficult to understand and might reflect chance findings. However, they are not in general disagreement with the effects found in the reaction times.

## Discussion

Our study investigated if peripheral cues can trigger retrieval of specific task sets from memory (i.e., task sets to search for specific target colors and color-associated response mappings). To this end, we created experimental settings in which similar or different response mappings were associated with differently colored targets. Our focused analyses revealed evidence for cue-based memory retrieval that broadens our current understanding of the role of cue-elicited memory in peripheral cueing.

## Evidence for cue-based retrieval of specific task sets

We investigated the interactions between task-set similarity and cue-target color congruence effects and made several interesting observations. First, our experiment revealed mixing and switch costs (the latter reflected in trial-by-trial target-color repetition effects), even in 2CS conditions. These costs are indicative of the maintenance of two separate task-sets, one for each of the two task-relevant colors used during two-color target search (see Moore and Weissman, 2010, 2011; Büsel et al., 2019). In addition, in line with our aim to introduce more different task-sets associated with each color, we observed increased mixing and trial-by-trial switch costs in 2CD compared to 2CS conditions.

Most critically, the findings supported that the top-down matching cues not only captured attention, but were also involved in the retrieval of color-associated task-sets from memory. First, cue-target color congruence (compared to -incongruence) facilitated search. This was expected if the cue color primed the perception of the target color (Irons et al., 2012) but also if the cue was involved in the retrieval of a color-associated task-set (Moore and Weissman, 2010, 2011; Büsel et al., 2019), which necessitated an updating of the activated task set under cue-target color-incongruent conditions (Adamo et al., 2010; Irons and Remington, 2013). Second, as predicted by cue-elicited task-set retrieval (cf. Frings et al., 2020) but not by feed-forward color priming, the cue-target color-congruence effect was stronger under different task-set than under similar task-set conditions. Third, a stronger cue-target color congruence effect under trial-by-trial target-color switch conditions could have indicated that cue-based retrieval in cue-target color congruent conditions partly counteracted the trial-by-trial target-color switch costs. However, in contrast to the interaction between task-set similarity and cue-target color congruence, the interaction between trial-by-trial target-color repetition and cue-target color congruence was not observed when we looked at relative effects (accounting for absolute reaction-time differences between 2CS and 2CD conditions). Thus, caution is advised when interpreting the second interaction, but the congruence-effect difference between 2CS and 2CD blocks alone demonstrated that mere feed-forward color priming alone was insufficient to explain the found effects (see also Ramgir and Lamy, 2021).

At the same time, we also observed important differences of the cue-target color congruence effect with matching cues in comparison to non-matching cues. First, in both 2CS and 2CD conditions, incongruent-matching cues delayed search relative to non-matching cues. Again, these results cannot be explained by the mere absence of color priming of targets through cues only because, if this were the case, there should have been no delays by matching-incongruent compared to non-matching cues. Thus, the matching-incongruent cues must have created additional interference, most likely through color-based retrieval of task sets that needed to be changed to process the targets. To note, this was found in 2CS conditions. Thus, there is evidence of the use of alternative task sets for different target colors even where the colors are not associated with different response mappings. However, in line with a stronger interference through incongruent cues that signaled an even more different task set, the delay by matching-incongruent relative to non-matching cues was stronger under 2CD than 2CS conditions. In addition, under 2CD conditions, the matching-congruent cue even facilitated search compared to the non-matching cue. This, however, was not found in the 2CS conditions. As the 2CS conditions allowed faster search, it could be that there was simply less room for further facilitation of search through cue-based retrieval of the correct task set under matching-congruent conditions.

In addition, in the current experiment, cue-based retrieval of task-set information about which colors to search for or about the color-associated response mappings had no modifying effect on validity effects under top-down matching conditions. This might appear as paradox in the light of the fact that sometimes participants must have held the wrong top-down task set in memory (i.e., a task-set for the color red) when a matching cue was presented (e.g., a matching green cue), for example, when a preceding trial's target color was different from the current trial's cue color (i.e., under trial-by-trial target color repetition/cue-target color-incongruent conditions). We believe that under such conditions, attention capture by target-similar cues and, hence, the validity effect, can occur during task-set retrieval itself—that is, the target-similar cue captures attention in its role as a task cue, too (cf. Mayr and Kliegl, 2003). Thus, similar validity effects under two- vs. one-color search tasks (cf. Kerzel and Witzel, 2019) might not be the most sensitive litmus criterion for the existence of color-specific task sets in contingent-capture experiments.

Our data, thus, also contribute to the ongoing debate about whether simultaneous search for multiple features is possible and efficient (e.g., Olivers et al., 2011; Irons et al., 2012; Büsel et al., 2019; Ort and Olivers, 2020; Kerzel and Grubert, 2021). While contingent capture was of similar size under cue-target color-congruent and cue-target color-incongruent conditions in the present experiment, the presence of mixing and switch costs replicated the findings of Büsel et al. (2019) and is indicative of the maintenance of two distinct task sets (Monsell, 2003).

## Conclusion

To sum up our results and interpretations, we have illustrated that top-down matching cues are involved in task-set retrieval from memory. Thus, the typical sequence of events with task-sets having to be activated before a top-down matching cue can capture attention is sometimes reversed, and the cue can trigger retrieval of a task-set corresponding to its features.

## Data availability statement

The raw data supporting the conclusions of this article will be made available by the authors, without undue reservation.

## Ethics statement

Ethical review and approval was not required for the study on human participants in accordance with the local legislation and institutional requirements. The participants provided their written informed consent to participate in this study.

## Author contributions

CB: Data curation, Formal analysis, Investigation, Methodology, Writing – original draft, Writing – review & editing. CV: Writing – original draft, Writing – review & editing. RS: Data curation, Writing – review & editing. PS: Supervision, Writing – review & editing. UA: Conceptualization, Investigation, Methodology, Supervision, Writing – original draft, Writing – review & editing.
