# Supplementary material for: Novel evidence for cue-based retrieval of top-down sets in spatial cueing
Source: Front Cognit. 2024 Mar 4;3:1305382. doi: 10.3389/fcogn.2024.1305382 (PMC13281089; doi:10.3389/fcogn.2024.1305382)
Supplement: Supplementary file 1 [file Data_Sheet_1.PDF]

Appendix A1

Influences of test environment

Here, we report influences of test environment. We decided to report these effects here because the effects reported below did not affect the overall pattern of results in a meaningful way and we wanted to present the main results relating to our research question as clearly and concisely as possible.

A1.1 Contingent-capture effects

Response Times. A significant main effect of test environment was found, with $F(1, 33) = 5.06$, $p = .031$, $\eta_p^2 = .13$. Participants responded 33 ms faster at home (394 ms) than in the laboratory (427 ms).

Error Rates. No main effect or interactions were found in error rates between test environment and any other variable (all *p*s > .35).

A1.2 Congruence and repetition

Response Times. The main effect of test environment was significant, with a 35 ms benefit for participants completing the experiment at home, $F(1, 33) = 4.74$, $p = .037$, $\eta_p^2 = .13$. In addition, in this analysis, the interaction between test environment, inter-trial target-color repetition, and task-set similarity was significant, $F(1, 33) = 4.91$, $p = .034$, $\eta_p^2 = .13$. First, looking at participants completing the experiment in the laboratory, we found small, yet significant trial-by-trial target-

color switch costs of 9 ms in the 2CS condition, $t(18) = 2.83$, $p = .01$, $d = 0.2$, and considerably larger target-color switch costs in the 2CD condition, 77 ms, $t(18) = 9.56$, $p < .001$, $d = 1.35$. A similar pattern was found for participants tested at home. However, target-color switch costs in the 2CS condition were slightly larger than in the laboratory, with 12 ms, $t(15) = 2.70$, $p = .016$, $d = 0.26$, and smaller in the 2CD condition, 59 ms, $t(15) = 7.28$, $p < .001$, $d = 0.88$. Student's *t*-tests revealed that target-color switch costs under neither condition differed significantly between test environments (both *p*s > .13).

Error Rates. A four-way interaction between test environment, validity, trial-by-trial target-color repetition, and task-set similarity was found, $F(1, 33) = 5.10$, $p = .031$, $\eta_p^2 = .13$. Post-hoc paired *t*-tests revealed that, for participants tested in the laboratory, trial-by-trial target-color switch costs were only found under 2CD conditions, both following valid cues, switch: 13.7% versus repetition: 7.5%, $t(18) = 2.46$, $p = .024$, $d = 0.69$, and invalid cues, switch: 14.9% versus repetition: 7.5%, $t(18) = 3.58$, $p = .002$, $d = 0.87$. For participants tested at home, a similar pattern was found, with no target-color switch costs under 2CS conditions. However, under 2CD conditions, target-color switch costs were only found following valid cues, switch: 15.2% versus repetition: 6.2%, $t(15) = 3.74$, $p = .002$, $d = 1.21$, but not ($p = .19$).

A1.3 Mixing costs

Response Times. There was no significant influence of test environment on mixing costs. Numerically, in the 2CS condition, mixing costs in the laboratory were 29 ms, $t(18) = 3.69$, $p = .002$, $d = 0.8$, and smaller with participants that were tested at home, 18 ms, $t(15) = 3.10$, $p = .007$, $d = 0.45$, but this difference was not significant ($p = .26$). The same pattern was found in the 2CD condition,

with numerically higher mixing costs found under laboratory conditions, 109 ms, $t(18) = 11.6$, $p < .001$, $d = 2.73$, than with participants tested at home, 103 ms, $t(15) = 12.11$, $p < .001$, $d = 2.02$. Again, the difference between these mixing costs was not significant ($p = .65$).

Error Rates. As in RTs, mixing costs did not differ significantly as a function of test environment, neither in 2CS conditions ($p = .13$), nor in 2CD conditions ($p = .37$).

Appendix A2

Full results of the supplementary analysis: Cue-target color congruency and non-matching cues

Response Times. The main effects of validity, trial-by-trial target-color repetition, and task-set similarity were significant with $F(1, 34) = 50.61$, $p < .001$, $\eta_p^2 = .60$, $F(1, 34) = 129.97$, $p < .001$, $\eta_p^2 = .79$, and $F(1, 34) = 442.6$, $p < .001$, $\eta_p^2 = .93$, respectively. These main effects were due to an overall validity effect of 10 ms and target-color switch costs of 37 ms. In addition, participants were 125 ms faster in 2CS conditions than in 2CD conditions. Target-color switch costs also differed significantly between task-set similarity conditions, $F(1, 34) = 97.2$, $p < .001$, $\eta_p^2 = .74$: In 2CS conditions, switch costs were small but reliable, 10 ms, $t(34) = 3.72$, $p < .001$, $d = 0.21$. Target-switch costs were considerably more pronounced in 2CD conditions, 69 ms, $t(34) = 11.68$, $p < .001$, $d = 1.06$.

Error Rates. Results for ERs mirror the results of RTs, as validity, trial-by-trial target-color repetition, and task-set similarity resulted in significant main effects, with $F(1, 34) = 5.95$, $p = .02$, $\eta_p^2 = .15$, $F(1, 34) = 26.2$, $p < .001$, $\eta_p^2 = .44$, and $F(1, 34) = 44.02$, $p < .001$, $\eta_p^2 = .56$, respectively. Participants committed more errors in invalid (8.9%) than in valid trials (7.7%), and more errors in

target-color switch (10%) than in target-color repetition trials (6.7%). Finally, 2CD conditions produced higher ERs (10.5%) than 2CS conditions (6%). Again, target-color switch costs differed significantly between task-set similarity conditions, $F(1, 34) = 26.2$, $p < .001$, $\eta_p^2 = .44$, with no significant target-color switch costs in 2CS conditions ($p = .77$), and significant target-color switch costs in 2CD conditions (repetition: 7.3% vs. switch: 13.7%), $t(34) = 6.02$, $p < .001$, $d = 1.04$.
